# HBV genotype-dependent association of HLA variants with the serodecline of HBsAg in chronic hepatitis B patients

**DOI:** 10.1038/s41598-023-27570-y

**Published:** 2023-01-07

**Authors:** Yu-Ju Chu, Hwai-I. Yang, Hui-Han Hu, Jessica Liu, Yu-Ling Lin, Chia-Ling Chang, Wen-Sheng Luo, Chin-Lan Jen, Chien-Jen Chen

**Affiliations:** 1 Genomics Research Center, Academia Sinica, 128 Academia Road, Section 2, Nankang, Taipei, Taiwan; 2 Institute of Microbiology and Immunology, National Yang-Ming University, Taipei, Taiwan; 3 Institute of Clinical Medicine, National Yang-Ming University, Taipei, Taiwan; 4 Graduate Institute of Medicine, College of Medicine, Kaohsiung Medical University, Kaohsiung, Taiwan; 5 School of Public Health, National Defense Medical Center, Taipei, Taiwan; 6 College of Medicine, Fu-Jen Catholic University, New Taipei, Taiwan

**Keywords:** Hepatitis B, Epidemiology

## Abstract

Seroclearance of hepatitis B surface antigen (HBsAg) is regarded as the functional cure for chronic hepatitis B (CHB). The relationship between human leukocyte antigen (HLA) variants, hepatitis B virus genotype, and longitudinal HBsAg serodecline remains to be explored. A total of 1735 HBeAg-seronegative CHB patients with genotype B or C infection of the community-based REVEAL-HBV cohort were genotyped for rs1710 (HLA-G) and rs2770 (HLA-B) using TaqMan assay. Cox proportional hazard regression and generalized linear mixed models were used to analyze the association of HLA genetic variants with the rate of HBsAg seroclearance and longitudinal HBsAg serodecline. Rs1710 G allele was differentially associated with the HBsAg seroclearance in genotype B [aRR (95% CI) = 0.74 (0.56–0.98)] and genotype C [aRR (95%CI) = 1.43 (1.08–1.88)] infection. Rs2770 G allele was associated with HBsAg seroclearance only in genotype B infection [aRR (95% CI) = 0.69 (0.52–0.91)]. The alleles associated with HBsAg seroclearance were significant predictors for the serodecline of HBsAg levels in an HBV genotype-dependent manner (genotype B infection: rs1710, *P* = 0.013; rs2770, *P* = 0.0081; genotype C infection: rs1710, *P* = 0.0452). Our results suggest both spontaneous HBsAg seroclearance and serodecline are modified by the interaction between HLA variants and HBV genotype.

## Introduction

Hepatitis B virus (HBV) infection has a significant impact on global health^1^. Seroclearance of HBV e antigen (HBeAg), HBV DNA, and surface antigen (HBsAg) are important milestones in the natural history of chronic HBV infection^2^. Deferred milestone transition and prolonged inflammation lead to liver cirrhosis, hepatic decompensation, and hepatocellular carcinoma (HCC) development with an estimated lifetime risk of 25–40% in the natural history of chronic hepatitis B (CHB)^2,3^. In Asian studies, CHB patients with genotype B infection have earlier HBeAg seroconversion, a lower tendency of disease progression, and a more favorable interferon-alpha treatment response compared to those with genotype C infection^4^. A higher HBsAg seroclearance rate was observed in genotype C than genotype B in our Risk Evaluation and Associated Liver Disease/Cancer-Hepatitis B Virus (REVEAL-HBV) cohort [RR (95% CI), 1.43 (1.13–1.81)] and a recent meta-analysis (C vs. B, 1.02% vs. 0.79%)^5^.

Spontaneous HBsAg seroclearance in chronic hepatitis B patients was considered a resolved HBV infection but a rare event in untreated individuals. The annual spontaneous HBsAg seroclearance rate was around 2%^6^. In patients whose HBsAg seroclearance occurs at ages younger than 50 years, the risk of cirrhosis and HCC was markedly reduced^7^. Spontaneous HBsAg seroclearance predictors include age, HBeAg serostatus, HBV genotype, and serum levels of HBV DNA and HBsAg^6,8,9^. In addition to viral factors, human leukocyte antigen (HLA) genes were identified as important genetic factors of HBV persistence by genome-wide association studies (GWAS)^10^ through regulating the adaptive immunity by presenting processed antigens for T cell recognition. Several 3’ untranslated region (UTR) variants in HLA-B, HLA-G, and HLA-DQA1 were significantly associated with spontaneous HBsAg seroclearance after adjustment for host and viral factors in our preliminary case–control study^11^. In this study, we aimed to investigate the impact of those identified HLA 3’UTR variants on spontaneous HBsAg seroclearance and serodecline in the REVEAL-HBV cohort.

## Results

The demographic features of study participants are listed in Table 1, most of the participants were under 50 years old (59.6%), male (62.5%), serum levels of alanine aminotransferase level (ALT) < 45 U/L (95.5%), HBV DNA < 1,000,000 copies/mL (93.8%), and HBsAg < 1000 IU/mL (55.8%), and genotype B infection (68.8%).

**Table 1:** Baseline characteristics of 1735 HBeAg-seronegative patients with genotype B or C chronic hepatitis B virus infection in the REVEAL-HBV cohort.

Characteristics	Number (%) (n = 1735)
**Age**	
30–39	507 (29.2)
40–49	528 (30.4)
50–59	528 (30.4)
60–69	172 (9.9)
**Gender**	
Female	650 (37.5)
Male	1085 (62.5)
**Cigarette smoking**	
No	1171 (67.5)
Yes	564 (32.5)
**Alcohol drinking**^**a**^	
No	1526 (88)
Yes	208 (12)
**Body mass index (kg/m**^**2**^**)**^**b**^	
< 30	1656 (95.6)
≥ 30	77 (4.4)
**Serum alanine aminotransferase level (U/L)**	
< 45	1657 (95.5)
≥ 45	78 (4.5)
**HBV genotype**^**d**^	
B	1193 (68.8)
C	542 (31.2)
**HBV DNA level (copies/mL)**	
≥ 1,000,000	108 (6.2)
100,000–999,999	229 (13.2)
10,000–99,999	453 (26.1)
300–9999	673 (38.8)
Undetectable	272 (15.7)
**HBsAg level (IU/mL)**	
≥ 1000	766 (44.2)
100–999	621 (35.8)
< 100	348 (20.1)

### Associations between HLA variants and HBsAg seroclearance by HBV genotype

Table 2 shows the incidence rate and multivariate-adjusted rate ratio (aRR) of HBsAg seroclearance by rs1710, rs2770, and HBV genotypes. By the additive model, the G allele was associated with a lower HBsAg seroclearance rate with a borderline significance in genotype B infection. The aRR (95% CI) for the G allele of 0.80 (0.61–1.04). However, in genotype C infection, G allele was significantly associated with a higher rate of spontaneous HBsAg seroclearance with the aRR (95% CI) of 1.42 (1.08–1.86) in the additive model. The effect of rs1710 on HBsAg seroclearance was modified by HBV genotype given the *P* value for the interactive effect of 0.0044 for the additive model.

**Table 2:** Incidence rate and multivariate-adjusted rate ratio of HBsAg seroclearance by HLA variants and HBV genotypes.

Variant genotype	No. of participants (%)	Case No. /Person-years of follow-up	Incidence rate (per 1000/yr)	Crude rate ratio (95% confidence interval)	*P* value^b^	Adjusted rate ratio (95% confidence interval)^a^	*P* value^b^
**rs1710 (HLA-G) C > G**							
*Genotype B (n* = *1189)*							
CC	431 (36)	51 /3361	15.17	1.00 (Referent)		1.00 (Referent)	
GC	550 (46)	54 /4658	11.59	0.75 (0.51–1.10)	0.135	0.63 (0.42–0.92)	0.018
GG	208 (17)	21 /1617	12.98	0.86 (0.52–1.42)	0.547	0.73 (0.44–1.23)	0.236
Additive model				0.88 (0.68–1.14)	0.338	0.80 (0.61–1.04)	0.095
*Genotype C (n* = *542)*							
CC	202 (37)	38 /1827	20.79	1.00 (Referent)		1.00 (Referent)	
GC	264 (49)	50 /2355	21.23	1.01 (0.66–1.54)	0.982	1.09 (0.71–1.69)	0.691
GG	76 (14)	25 /670	37.34	1.98 (1.20–3.29)	0.008	2.13 (1.27–3.56)	0.004
Additive model				1.36 (1.03 1.78)	0.028	1.42 (1.08–1.86)	0.012
**rs2770 (HLA-B) A > G**							
*Genotype B (n* = *1139)*							
AA	539 (47)	62 /4300	14.42	1.00 (Referent)		1.00 (Referent)	
AG	476 (42)	48 /3874	12.39	0.84 (0.58–1.23)	0.375	0.76 (0.51–1.11)	0.156
GG	124 (11)	8 /1061	7.54	0.49 (0.24–1.03)	0.061	0.40 (0.19–0.85)	0.017
Additive model				0.76 (0.58–1.01)	0.060	0.69 (0.51- 0.92)	0.011
*Genotype C (n* = *518)*							
AA	228 (44)	52 /2023	25.7	1.00 (Referent)		1.00 (Referent)	
AG	230 (44)	43 /2111	20.37	0.75 (0.50–1.13)	0.166	0.82 (0.54–1.26)	0.366
GG	60 (12)	11 /545	20.18	0.71 (0.37–1.37)	0.305	0.74 (0.38–1.44)	0.377
Additive model				0.81 (0.60–1.08)	0.154	0.85 (0.63–1.14)	0.280

^a^Adjusted for age, gender, body mass index, and levels of HBV DNA and HBsAg.

^b^Estimated by Cox proportional hazards model.

^#^*P* value for interaction between HBV genotype and rs1710 is 0.0044 (additive model). *P* value for interaction between HBV genotype and rs2770 is 0.3588 (additive model).

The effect of the rs2770 genotype was more profound in genotype B HBV-infected patients (Table 2). The G allele was significantly associated with a lower HBsAg seroclearance rate given aRR (95% CI) of 0.69 (0.51–0.92) in the additive model. No such association was seen in genotype C infection.

### Predictors of HBsAg seroclearance by HBV genotype

In genotype B infection, cigarette smoking, low serum levels of HBV DNA and HBsAg were significantly associated with an increased rate of HBsAg seroclearance while rs1710 G allele [aRR (95% CI) = 0.74 (0.56–0.98)] and rs2770 G allele [aRR (95% CI) = 0.69 (0.52–0.91)] were associated with a significantly decreased rate of spontaneous HBsAg seroclearance in the additive model (Table 3). In genotype C infection, only low serum HBsAg levels and G allele of rs1710 were significant predictors of spontaneous HBsAg seroclearance (Table 4). The multivariate-adjusted RR (95% CI) for G allele of rs1710 was 1.43 (1.08–1.88) in the additive model.

**Table 3:** Multiple predictors for the seroclearance of HBsAg in HBeAg-seronegative chronic hepatitis B patients with genotype B HBV infection.

Baseline characteristics	Crude rate ratio (95% CI)	*P* value^c^	Adjusted rate ratio (95% CI)	*P* value^c^
**Age**				
< 40	1.00 (Referent)			
40–49	0.90 (0.55–1.46)	0.660		
50–59	1.22 (0.77–1.93)	0.391		
≥ 60	1.99 (1.11–3.59)	0.022		
**Gender**				
Female	1.00 (Referent)			
Male	0.62 (0.41–0.94)	0.025		
**Cigarette smoking**				
No	1.00 (Referent)		1.00 (Referent)	
Yes	1.34 (0.93–1.92)	0.113	1.45 (1.01–2.09)	0.046
**Alcohol drinking**				
No	1.00 (Referent)			
Yes	0.92 (0.53–1.61)	0.779		
**Body mass index (Kg/m**^**2**^**)**^**a**^				
< 30	1.00 (Referent)			
≥ 30	2.02 (1.05–3.86)	0.034		
**Serum alanine *aminotransferase* level (U/L)**				
< 45	1.00 (Referent)			
≥ 45	1.22 (0.50–2.99)	0.665		
**HBV DNA level (copies/mL)**				
≥ 10,000	1.00 (Referent)		1.00 (Referent)	
300–9999	1.83 (1.16–2.87)	0.009	1.13 (0.70–1.83)	0.610
Undetectable	4.54 (2.84–7.26)	< .0001	2.64 (1.58–4.40)	0.000
**HBsAg level (IU/mL)**				
≥ 1000	1.00 (Referent)		1.00 (Referent)	
100–999	4.86 (2.27–10.39)	< .0001	5.03 (2.35–10.78)	< .0001
< 100	17.64 (8.49–36.65)	< .0001	15.24 (7.20–32.26)	< .0001
**rs1710**				
Recessive model	0.94 (0.57–1.53)	0.797		
Additive model	0.86 (0.66–1.12)	0.256	0.74 (0.56–0.98)	0.035
**rs2770**				
Recessive model	0.53 (0.26–1.09)	0.086		
Additive model	0.77 (0.58–1.01)	0.062	0.69 (0.52–0.91)	0.009

^a^One participant was missing for the data of body mass index.

^b^Estimated by Cox proportional hazards model.

**Table 4:** Multiple predictors for the seroclearance of HBsAg in HBeAg-seronegative chronic hepatitis B patients with genotype C HBV infection.

Baseline characteristics	Crude rate ratio (95% CI)	*P* value^c^	Adjusted rate ratio (95% CI)	*P* value^c^
**Age**				
< 40	1.00 (Referent)			
40–49	1.54 (0.87–2.74)	0.142		
50–59	1.69 (0.95–3.00)	0.075		
≥ 60	2.63 (1.31–5.29)	0.007		
**Gender**				
Female	1.00 (Referent)			
Male	0.97 (0.66–1.43)	0.878		
**Cigarette smoking**				
No	1.00 (Referent)			
Yes	1.64 (1.08–2.47)	0.020		
**Alcohol drinking**^**a**^				
No	1.00 (Referent)			
Yes	1.34 (0.78–2.32)	0.293		
**Body mass index (Kg/m**^**2**^**)**^**b**^				
< 30	1.00 (Referent)			
≥ 30	1.68 (0.82–3.46)	0.160		
**Serum alanine *aminotransferase* level (U/L)**				
< 45	1.00 (Referent)			
≥ 45	0.87 (0.41–1.88)	0.728		
**HBV DNA level (copies/mL)**				
≥ 10,000	1.00 (Referent)			
300–9999	1.33 (0.82–2.16)	0.246		
Undetectable	2.75 (1.65–4.60)	0.000		
**HBsAg level (IU/mL)**				
≥ 1000	1.00 (Referent)		1.00 (Referent)	
100–999	3.57 (2.18–5.84)	< .0001	3.66 (2.23–6.00)	< 0.0001
< 100	12.62 (7.79–20.42)	< .0001	12.84 (7.93–20.79)	< 0.0001
**rs1710**				
Recessive model	2.12 (1.35–3.33)	0.001		
Additive model	1.37 (1.03–1.81)	0.029	1.43 (1.08–1.88)	0.012
**rs2770**				
Recessive model	0.82 (0.44–1.53)	0.532		
Additive model	0.81 (0.60–1.08)	0.154		

^a^One participant was missing for the data of habitual drinking status.

^b^One participant was missing for the data of body mass index.

^c^Estimated by Cox proportional hazards model.

### HLA variants and serodecline of HBsAg levels by HBV genotype

**Figure 1:**
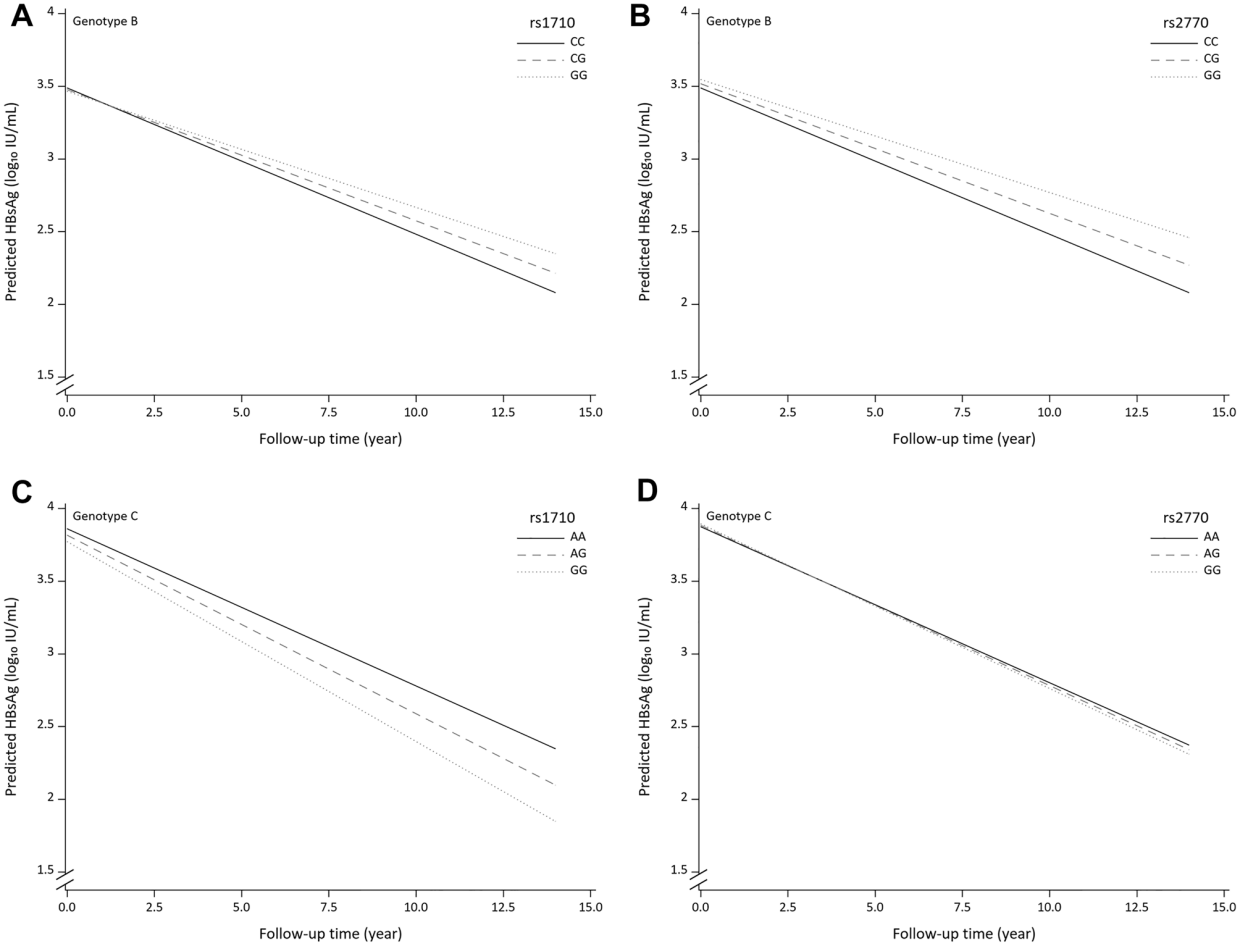
Longitudinal change in serum HBsAg levels during the 14-year follow-up period in HBeAg-seronegative chronic hepatitis B patients. (**A**) Among patients with genotype B HBV infection by rs1710 genotypes (*P* = 0.013). (**B**) Among patients with genotype B HBV infection by rs2770 genotypes (*P* = 0.0081). (**C**) Among genotype C HBV infection by rs1710 genotypes (*P* = 0.0452). (**D**) Among genotype C HBV infection by rs1710 genotypes (*P* = 0.6977). *P* values were estimated by the generalized linear mixed model (GLIMMIX) with the adjustment of other significant predictors of HBsAg seroclearance in proportional hazards models.

There was no significant association between genotypes of rs1710 and rs2770 and the baseline serum levels of HBV DNA and HBsAg, except that the rs1710 G allele in genotype C infection and rs2770 A allele in genotype B infection were associated with higher baseline serum levels of HBV DNA (Supplementary Table 2). To determine whether these two HLA variants have an impact on the long-term serodecline of HBsAg levels, we further examined the relationship between the decline in serum HBsAg levels and the genotypes of rs1710 and rs2770 (Fig. 1). In genotype B infection, the G allele**s** of both rs1710 and rs2770 were significantly associated with higher serum HBsAg levels (i.e., lower serodecline) under the additive model given the *P* value of 0.013 (Fig. 1A) and 0.0081 (Fig. 1B) for rs1710 and rs2770, respectively. In genotype C infection, the G allele of rs1710 was significantly associated with lower serum HBsAg levels (i.e., higher serodecline) under the additive model (Fig. 1C; *P* value = 0.0452); the rs2770 variant was not significantly associated with the serodecline in HBsAg levels during the follow-up period (Fig. 1D; *P* value = 0.6977).

## Discussion

In this long-term follow-up cohort study, the G allele of both rs1710 and rs2770 was associated with a decreased HBsAg seroclearance rate and lower serodecline in HBsAg levels over the follow-up period in CHB patients infected with HBV genotype B. However, the rs1710 G allele was significantly associated with an increased HBsAg seroclearance rate and higher serodecline in HBsAg levels over time in those infected with HBV genotype C.

Our previous studies suggest a chronological order of seroclearance of HBV viral markers from HBeAg seroclearance, HBV DNA seroclearance to HBsAg seroclearance in CHB patients without antiviral treatment^2,6^. There was no association between rs1710 or rs2770 genotype and HBeAg seroconversion nor HBV DNA undetectable status in this cohort study (Supplementary Table 3), suggesting that these variants specifically contribute to the HBsAg seroclearance. The preferential association of rs2770 genotype with HBsAg seroclearance in genotype B infection in this study echoes the finding that several HLA-DP polymorphisms were associated with HBV natural clearance preferentially in genotype B infection^12^. In addition to validating the findings that HLA SNPs were associated with HBV clearance/persistence^10,13^, this is the first study to prove the concept that the HLA variants may have a different impact on HBsAg seroclearance by HBV genotype in a large untreated prospective HBV cohort.

Functional analysis of HLA-DPB1 suggests that the protective and susceptible alleles have distinct amino acid residues in the peptide-binding pocket 1 which determines the antigen-binding specificities^14^. Besides, genetic variants in the regulatory region may result in differential post-transcriptional regulation and further change the mRNA expression level^15,16^. For example, the reported susceptible alleles for CHB, rs3077 and rs9277535, located within the 3’ UTR of HLA genes were found to be strongly associated with the reduced mRNA expression level of HLA-DPA1 and HLA-DPB1, respectively^17^. Apart from the direct impact on the mRNA expression level, the 3’UTR variant may also be in linkage disequilibrium (LD) with causal variants in other regions such as the sequences coding for α or β chain of HLA molecules responsible for peptide binding specificity. Hence, HLA variants may lead to variable HLA expression levels and antigen presentation efficiencies, influencing the clinical outcome of chronic viral infection including CMV, HIV, HPV, and HBV^15,16,18–20^.

HLA-G exerts its inhibitory function on antigen-presenting cells, natural killer cells, and B and T lymphocytes by interacting with the inhibitory receptors^21^. The increased HLA-G expression^22,23^, in which CD4^+^CD25^+^FoxP3^+^ T regulatory cells and HLA-G^+^ CD4^+^ monocytes also significantly increased^23^, was found to be associated with CHB persistence/progression^23^. The variability of the HLA-G genes and the variants with high frequency were identified in the 3’UTR including rs1063320 and rs1710^24^. Polymorphisms in the HLA-G 3’UTR may influence HLA-G expression levels^25^. While a computational and functional study demonstrating that rs1063320 (+ 3142, C > G) variant affects the binding of miR-148a, miR-184b, and miR-152 and hence the expression level of HLA-G^26^, another functional study failed to validate this regulatory effect^27^. The rs1710 polymorphism was predictive of the miRNA binding^28^, but the impact of the rs1710 (+ 3010, G > C) variant on the HLA-G expression remains uncertain. We performed in silico analysis to predict the potential effect of HLA variants on the miRNA binding to HLA 3’UTR sequences using the PolymiRTS Database 3.0 (http://compbio.uthsc.edu/miRSNP^29^ and the miRNASNP 3.0 database (http://bioinfo.life.hust.edu.cn/miRNASNP^30^. The results of in silico analysis for predicting gain or loss of miRNA-binding sites were shown in Supplementary Table 4.

The contradictory effect of the rs1710 G allele on HBsAg seroclearance in genotype B and C infection is intriguing. We used the LDlink (https://ldlink.nci.nih.gov/?tab=ldmatrix) to analyze the matrix of pairwise LD statistics of rs1710, rs1063320, and HLA-G exonic variants based on the data of Han Chinese in Beijing and Southern Han Chinese populations^24^. All the reported exonic variants in high LD with rs1710 including rs1630185, rs1130355, and rs1130363 result in synonymous changes. Because rs1710 and rs1063320 polymorphism may influence the mRNA expression level by altering the mRNA-miRNA interaction, rs1710 and rs1063320 may confer the HBV genotype-dependent association on HBsAg seroclearance by regulating the function of HLA-G targeting immune cells with HBV genotype-dependent effect.

The variability in the HLA-B gene is not well investigated as the HLA-G gene, and rs2770 was rarely reported in previous studies. In our in silico analysis (Supplementary Table 4), the rs2770 polymorphism was predicted to alter the binding affinity of hsa-miR-142-5p to HLA-B mRNA. Interestingly, hsa-miR-142-5p expression level was found to be significantly reduced in HBeAg-negative CHB patients compared to that in inactive carriers^31^. HLA-B encodes the class I HLA presenting the foreign antigen to activate cytolytic CD8^+^ T cells and mount the adequate immune response. The increased frequency of functional cytolytic CD8^+^ T cells may lead to HBsAg seroclearance in HBeAg-negative CHB patients^32^. HLA-B allele type has also been implicated in the HBV spontaneous clearance^33^. However, whether rs2770 is in LD with other variants in the coding region altering the viral peptide epitope binding is unknown.

The HBV genotype-dependent association of HLA variants with HBsAg serodecline is interesting but the underlying mechanism remains to be deciphered. In the case of HLA-G, we hypothesize that HLA-G suppresses the activity of immune cells such as CD8^+^ T-cells which recognize certain epitopes specifically presenting in genotype B or C HBV^34^. Hence, alteration in the HLA-G expression by the change of mRNA-miRNA interaction may result in different impacts on downstream immune responses and disease outcomes. Besides, it is also plausible that these HLA variants are in genetic linkage with other causal variants located within HLA loci encoding the antigen-binding peptides. Protective or pathogenic immune responses are mounted depending on the interaction between the allotypes and certain epitope peptides recognized in specific HBV genotypes and therefore resulted in HBV genotype-dependent effect. However, more molecular and functional studies are needed to investigate the underlying mechanisms for the HBV genotype-dependent effect observed in this study.

Apart from the numerical difference in HBsAg seroclearance between HBV genotype B and genotype C infected CHB patients in recent meta-analysis studies^5^, the incidence rate of spontaneous HBsAg seroclearance was significantly higher in genotype C than B [17.8 versus 12.0 cases per 1,000 person-years, RR (95% CI) of 1.43 (1.13–1.81)] in REVEAL-HBV longitudinal cohort. In this study, we found significant HBV genotype-dependent associations between HBsAg seroclearance and rs1710 and rs2770 genotypes. This finding is worthy to be validated by another independent cohort before incorporation into the scoring system for predicting HBsAg seroclearance and risk stratification^8^.

Due to the longer follow-up interval (6–12 months) of the participants in this community-based cohort compared to patients from the hospital-based cohort (1–2 weeks) and the lack of data on the level of fibrosis, we were unable to assess the correlation between the HLA variants and other clinical parameters such as hepatic flare and fibrosis. However, no significant association of rs1710 and rs2770 with the risk of HCC (data not shown) was observed in this population. Considering HBsAg seroclearance being just one of the predictors for the risk of HCC^35^ and the complicated interaction between host and viral factors shaping the disease progression of CHB, it is not surprising that these HLA variants were not directly associated with the risk of HCC. Nevertheless, whether these variants HLA variants have potential impacts on other disease outcomes deserves further investigation.

There are several advantages of using the cohort study to investigate the association between HLA 3’UTR polymorphisms and HBsAg seroclearance and serodecline. Firstly, the cohort study may reduce the bias resulting from the case–control studies; secondly, the HBsAg seroclearance incidence rate and the rate ratio of groups carrying different variant genotypes were assessable in the cohort study; lastly, our cohort study collected serial blood samples of the participants during the follow-up period enabling the analysis of the variant genotype’s impact on and the longitudinal serodecline of HBsAg. By repeated measurement of HBsAg, two HLA variants, rs1710 and rs2770, contribute to the serodecline in HBsAg level during longitudinal follow-up in an HBV genotype-dependent manner.

In conclusion, this is the first study demonstrating the impact of HLA variants on spontaneous HBsAg seroclearance and serodecline differed by HBV genotype in an untreated prospective cohort. More studies on HLA polymorphisms other than 3’UTR are required to clarify the interactive effect of HLA and HBV genotype on the clinical outcomes of CHB.

## Methods

### Study cohort

The study cohort included anti-HCV-seronegative and HBeAg-seronegative participants who had complete data of baseline and follow-up serum HBsAg levels from the REVEAL-HBV study, which was approved by the Institutional Review Board of the Academia Sinica, Taipei, Taiwan^36^. The study was conducted in accordance with the principles stated in the Declaration of Helsinki. This community-based study was launched in 1991–1992, and 23,820 residents aged 30 to 65 years were recruited from seven townships across Taiwan. Each participant provided written informed consent for a questionnaire interview by trained public health nurses, health examination, and biospecimen collection at study entry and follow-up as described in our earlier studies^36^. A total of 4,155 participants were HBsAg-seropositive and free of HCC at study entry. These CHB patients received follow-up examinations of abdominal ultrasonography and serological test every 6–12 months until June 30, 2004. HBsAg-seropositive participants who were HBeAg-seropositive (n = 457), antibody against HCV (anti-HCV)-seropositive (n = 218), without baseline or follow-up serum HBsAg levels (n = 890), or without DNA samples available for TaqMan SNP genotyping assay (n = 154) were excluded. CHB patients with undeterminable HBV genotype due to lack of adequate serum samples (n = 100) or low HBV viral load (n = 593) and those coinfected with genotype B and C HBV (n = 70) were further excluded from the association analysis. A total of 1,735 HBeAg-seronegative patients with genotype B or C HBV infection were included in this study.

### Data collection and serological tests

HBeAg and HBsAg serostatus were detected by radioimmunoassay (Abbott Laboratories). Anti-HCV was detected by enzyme immunoassay using a second-generation test kit (Abbott Laboratories). Serum ALT levels were measured by the serum chemistry autoanalyzer (Model 736, Hitachi Co.) using commercial reagents (Biomerieux). Serum HBV DNA levels were assayed by the COBAS Amplicor HBV monitor test kit (Roche Diagnostics). Serum HBsAg levels were quantified by the Elecsys HBsAg II Quant assay (Roche Diagnostics). HBV genotype was determined by melting curve analysis in participants with detectable serum HBV DNA levels.

### TaqMan genotyping assay

Among nine MiSeq sequencing-determined variants in HLA-B, HLA-G, and HLA-DQA1 which were significantly associated with spontaneous HBsAg seroclearance in our preliminary matched case–control study^11^, the TaqMan genotyping assay was successfully designed only for rs1710 (C > G), rs1063320 (G > C), and rs2770 (A > G) due to the highly polymorphic sequences in the flanking region of most other variants. Genomic DNA was extracted from buffy coat using QIAamp Blood Mini kit (QIAgen) and genotyped for the rs1710, rs1063320, and rs2770 polymorphisms by TaqMan SNP genotyping assays (Applied Biosystems) on an Applied Biosystems 7900 HT Fast Real-Time PCR System according to manufacturer’s protocols. The sequences of TaqMan probes and primers are shown in Table, Supplementary Table 1. All samples were assayed blinded to the HBsAg serostatus and serological test results. Four samples were randomly selected to repeat in each 384-well assay. The gold-standard Sanger sequencing was used to confirm the TaqMan genotyping results of 23 samples, and the concordance rate between TaqMan genotyping and Sanger sequencing was 100%. The genotyping rate for rs1710, rs1063320**,** and rs2770 was 99.53%, 98.17%**,** and 94.79%, respectively. Because rs1710 and rs1063320 were highly linked (r^2^ = 0.87), the analysis of associations with HBsAg seroclearance and the HBsAg serodecline was carried out only for rs1710 and rs2770.

### Statistical analysis

Incidence rates of HBsAg seroclearance per 1,000 person-years were estimated for genotypes of rs1710 and rs2770. Cox proportional hazards model was used to estimate the crude and adjusted rate ratios (RRs) with 95% confidence intervals (CIs). The stepwise selection was performed to determine the predictors for spontaneous HBsAg seroclearance. The generalized linear mixed model (GLIMMIX) was used to predict the longitudinal serodecline in HBsAg levels over time with the adjustment of other significant predictors of HBsAg seroclearance in proportional hazards models. Locally weighted scatterplot smoothing (LOESS) was used to generate the smoothed scatterplot of the relationship between predicted follow-up HBsAg serum level and follow-up time. SAS 9.4 was employed for data management and statistical analysis.

### Ethics approval statement

This study was approved by the Institutional Review Board of the Academia Sinica, Taipei, Taiwan. The study was conducted in accordance with the principles stated in the Declaration of Helsinki.

### Patient consent statement

Each participant in this study provided written informed consent for a questionnaire interview by trained public health nurses, health examination, and biospecimen collection at study entry and follow-up.

### Permission to reproduce material from other sources

There’s no material reproducing from other sources in this study.

## Supplementary Information

Supplementary Information.

## Data Availability

The datasets used and/or analysed during the current study available from the corresponding author on reasonable request.

## Abbreviations

HBV: Hepatitis B virus
CHB: Chronic hepatitis B
HBeAg: HBV e antigen
HBsAg: HBV surface antigen
RR: Rate ratio
CI: Confidence interval
HLA: Human leukocyte antigen
UTR: Untranslated region
ALT: Alanine aminotransferase
SNP: Single nuclear polymorphism
