# Supplementary material for: HBV genotype-dependent association of HLA variants with the serodecline of HBsAg in chronic hepatitis B patients
Source: Sci Rep. 2023 Jan 7;13:359. doi: 10.1038/s41598-023-27570-y (PMC9825396; doi:10.1038/s41598-023-27570-y)
Supplement: Supplementary file 1 — Supplementary Information. [file 41598_2023_27570_MOESM1_ESM.pdf]

Supplementary Table 1. Sequences of primer and probe for TaqMan genotyping assay of HLA 3'UTR variants

HLA 3'UTR variants	Sequence (5'-3')	
rs1710	Primer	F: CCTTTGTGACTTCAAGAACCCTGA
		R: GAAGGAATGCAGTTCAGCATGAG
	Probe	VIC-TCTCTGCAGAAAGAG-NFQ
		FAM-TCTCTGCACAAAGAG-NFQ
rs1063320	Primer	F: CCTTCCCCAATCACCTTTCCT
		R: TCTCTCAAATTTCAGGAATCTTCTCCTT
	Probe	VIC-TTATAGCTCAGTGGACCACA-NFQ
		FAM-TAGCTCAGTGCACCACA-NFQ
rs2770	Primer	F: AAGAAGTTGCAGCTCAGT
		R: TGTGTTTCCTCCCCAGT
	Probe	VIC-TCTGTCTCAACTTTATGTG-NFQ
		FAM-TCTGTCTCAACTTTACGTG-NFQ

Supplementary Table 2. Association between genotypes of rs1710 and rs2770 and baseline serum levels of HBV DNA and HBsAg in 1655 HBeAg-seronegative CHB patients genotyped for both variants by HBV genotype

	Genotype B (n=1137)			Genotype C (n=518)		
	No (%)	No (%)	p value	No (%)	No (%)	p value
rs1710 (HLA-G) C>G	CC+CG[ǂ]	GG		CC+CG	GG[ǂ]	
HBV DNA level (copies/mL)						
Undetectable	131 (14.0)	25 (12.3)	0.056	93 (21.0)	13 (17.3)	0.174
300-9999	341 (36.5)	67 (33.0)		203 (45.8)	30 (40.0)	
10000-99999	256 (27.4)	60 (29.6)		96 (21.7)	18 (24.0)	
100000-999999	140 (15.0)	44 (21.7)		29 (6.6)	5 (6.7)	
≥100000	66 (7.1)	7 (3.5)		22 (5.0)	9 (12.0)	
Mean± SD (log)	4.31 ± 1.15	4.40 ± 1.07	0.356	4.00 ± 1.05	4.35 ± 1.41	0.069
HBsAg level (IU/mL)						
<100	210 (22.5)	44 (21.7)	0.360	62 (14.0)	10 (13.3)	0.938
100-999	395 (42.3)	77 (37.9)		107 (24.2)	17 (22.7)	
≥1000	329 (35.2)	82 (40.4)		274 (61.9)	48 (64.0)	
Mean± SD (log)	2.51 ± 0.99	2.52 ± 1.11	0.891	2.93 ± 1.24	2.91 ± 1.27	0.896
rs2770 (HLA-B) A>G	AA+AG[ǂ]	GG		AA+AG[ǂ]	GG	
HBV DNA level (copies/mL)						
Undetectable	132 (13.0)	24 (19.4)	0.014	95 (20.7)	11 (18.3)	0.910
300-9999	357 (35.2)	51 (41.1)		203 (44.3)	30 (50.0)	
10000-99999	282 (27.8)	34 (27.4)		103 (22.5)	11 (18.3)	
100000-999999	176 (17.4)	8 (6.5)		30 (6.6)	4 (6.7)	
≥100000	66 (6.5)	7 (5.7)		27 (5.9)	4 (6.7)	
Mean± SD (log)	4.36 ± 1.13	4.08 ± 1.16	0.022	4.06 ± 1.11	3.97 ± 1.19	0.574
HBsAg level (IU/mL)						
<100	229 (22.6)	25 (20.2)	0.560	66 (14.4)	6 (10.0)	0.650
100-999	415 (41.0)	57 (46.0)		109 (23.8)	15 (25.0)	
≥1000	369 (36.4)	42 (33.9)		283 (61.8)	39 (65.0)	
Mean± SD (log)	2.51 ± 1.01	2.50 ± 1.02	0.871	2.90 ± 1.28	3.13 ± 0.89	0.074

[ǂ] Genotypes of variants with a higher incidence rate of HBsAg seroclearance.

Supplementary Table 3. Association of genotypes of rs1710 and rs2770 with the seroclearance of HBeAg and HBV DNA in CHB patients

HLA variant	Crude rate ratio (95% CI)	p value	Adjusted rate ratio (95% CI)	p value
Seroclearance of HBeAg				
rs1710 (HLA-G) C>G				
Recessive model	0.98 (0.88-1.08)	0.660	0.98 (0.89-1.09)	0.762
Additive model	0.99 (0.94-1.05)	0.756	1.00 (0.94-1.05)	0.848
rs2770 (HLA-B) A>G				
Recessive model	0.98 (0.87-1.11)	0.760	0.98 (0.87-1.11)	0.781
Additive model	0.98 (0.93-1.04)	0.484	0.98 (0.93-1.04)	0.531
Seroclearance of HBV DNA				
rs1710 (HLA-G) C>G				
Recessive model	0.95 (0.81-1.12)	0.562	1.06 (0.89-1.25)	0.517
Additive model	1.01 (0.93-1.10)	0.793	1.04 (0.95-1.14)	0.376
rs2770 (HLA-B) A>G				
Recessive model	1.09 (0.91-1.31)	0.335	0.96 (0.80-1.15)	0.654
Additive model	1.04 (0.961.14)	0.351	0.96 (0.87-1.05)	0.317

[a] For association with seroclearance of HBeAg, the rate ratio was adjusted for HBV genotype and serum level of HBV DNA at study entry among HBeAg-seropositive patients. For association with seroclearance of HBV DNA, the rate ratio was adjusted for HBV genotype, and serum levels of HBV DNA and HBsAg at study entry among HBeAg-seronegative patients.

Supplementary Table 4. *In silico* analysis results for potential gain or loss of miRNA binding sites to HLA 3'UTR regions by HLA variants

Gene	Variant ID	Ref/Alt	Predicted miRID	miRNASNP v3.0	PolymiRTS Database 3.0
HLA-B	rs2770	G>A	hsa-miR-142-5p	Create miRNA binding site	Create miRNA binding site
			hsa-miR-5589-3p	Create miRNA binding site	
			hsa-miR-5590-3p	Create miRNA binding site	Create miRNA binding site
HLA-G	rs1710	G>C	hsa-miR-1184	Create miRNA binding site	
			hsa-miR-10394-5p	Create miRNA binding site	
			hsa-miR-3678-3p	Create miRNA binding site	
			hsa-miR-1273h-3p	Create miRNA binding site	
			hsa-miR-1205	Create miRNA binding site	
			hsa-miR-3158-5p	Create miRNA binding site	
			hsa-miR-298	Create miRNA binding site	
			hsa-miR-17-3p	Create miRNA binding site	
			hsa-miR-3130-3p	Disrupt miRNA binding site	
			hsa-miR-4719	Disrupt miRNA binding site	
			hsa-miR-4793-3p	Disrupt miRNA binding site	
HLA-G	rs1063320	C>G	hsa-miR-500a-3p	Create miRNA binding site	
			hsa-miR-148b-3p	Create miRNA binding site	
			hsa-miR-152-3p	Create miRNA binding site	
			hsa-miR-454-3p	Create miRNA binding site	
			hsa-miR-767-5p	Create miRNA binding site	Create miRNA binding site
			hsa-miR-130a-3p	Create miRNA binding site	
			hsa-miR-4295	Create miRNA binding site	
			hsa-miR-301a-3p	Create miRNA binding site	
			hsa-miR-148a-3p	Create miRNA binding site	
			hsa-miR-6776-3p	Create miRNA binding site	
			hsa-miR-301b-3p	Create miRNA binding site	
			hsa-miR-5190	Create miRNA binding site	
			hsa-miR-3138	Disrupt miRNA binding site	
			hsa-miR-194-3p	Disrupt miRNA binding site	
			hsa-miR-4800-5p	Disrupt miRNA binding site	Disrupt miRNA binding site
			hsa-miR-4800-5p	Disrupt miRNA binding site	
			hsa-miR-3619-3p	Disrupt miRNA binding site	Disrupt miRNA binding site
			hsa-miR-12115	Disrupt miRNA binding site	
			hsa-miR-4535	Disrupt miRNA binding site	
			hsa-miR-5693	Disrupt miRNA binding site	
			hsa-miR-6849-5p	Disrupt miRNA binding site	
			hsa-miR-3681-5p	Disrupt miRNA binding site	
			hsa-miR-4776-5p	Disrupt miRNA binding site	Disrupt miRNA binding site
